# Exploring the Application of BIM Technology in the Whole Process of Construction Cost Management with Computational Intelligence

**DOI:** 10.1155/2022/4080879

**Published:** 2022-09-09

**Authors:** Dan Tang, Kongling Liu

**Affiliations:** School of Architecture and Engineering, Hunan Institute of Engineering, Xiangtan, Hunan 411100, China

## Abstract

The construction industry is a labor-intensive industry in China. In recent years, as people's living standards have risen, so have their requirements for the functionality, appearance, and comfort of buildings. The amount of information attached to construction projects is also increasing, especially for some modern large-scale construction projects. Due to the long construction period and a large amount of information in the construction process, the difficulty of project engineering management has also increased significantly. At the same time, with the gradual development and improvement of China's market economy system, the prices of labor and materials in the construction industry continue to rise, and the huge consumption of energy and resources no longer meets the strategic requirements of sustainable development. Therefore, construction project cost management is facing a huge impact, the actual effect of construction project cost management work is not good, and the phenomenon of uncontrolled construction project investment commonly occurs. The use of BIM software to build a building information model can integrate information from all stages of the project and facilitate the participation and cooperation of all project entities. Cost management is carried out before the actual construction of the project, thus realizing the whole process of cost management and effectively avoiding the occurrence of actual costs exceeding the budget after the project is completed. The study analyses the suitability of applying BIM technology to the whole process of construction project cost management with computational intelligence and explains the value and advantages of BIM in the whole process of construction project cost management. On this basis, this research provides a specific analysis of the application of BIM technology in the process of cost management at various stages of the whole process of construction projects and thus puts forward suggestions that can solve the obstacles that may be encountered in the whole process of cost management of construction projects in China.

## 1. Introduction

As one of the oldest industries in the world, the construction industry has always played a very important role in the history of human development. The construction industry has an irreplaceable position in China's national economic growth and social and economic development and has become an important pillar industry of the national economy [1]. In recent years, influenced by the national macrocontrol policies, the development of China's construction industry has gradually slowed down, but the construction industry output value in China's national economy still occupies a large proportion, and the proportion is increasing year by year trend. For a long time, the construction industry has been a labor-intensive industry in China, and the phenomenon of low efficiency and high construction costs is more common [2]. As living standards improve, people are demanding more and more information about the function, appearance, and comfort of buildings, and the amount of information attached to construction projects is increasing [3]. In the course of the actual construction of a project, changes are often made due to design failures or communication problems between the parties involved, especially for modern large-scale construction projects with large investment scales. Due to the large number of construction units involved and the long construction cycle, the difficulty of project management is greatly increased, and the problem of cost management is particularly acute [4]. However, because of the lack of overall planning for the whole life cycle of China's construction industry management, the attention of project participants is still only on a certain stage of the life cycle and a certain partial business. For instance, the traditional cost management model is unable to achieve information sharing throughout the construction life cycle [5]. Therefore, in order to ensure the effectiveness of engineering projects and reduce the waste of resources, it is necessary to improve the engineering management model, especially cost management which plays an important role in engineering management [6]. The effective use of information technology in the processing of various relevant information to achieve the whole process of project cost management can fundamentally reduce engineering changes and delays, thus reducing costs and maximizing the benefits of the project.

The three main objectives of construction project management are cost [7], quality [8], and duration [9]. With the continuous development of modern construction material technology, quality and duration are no longer universal issues in the construction industry. In the competitive environment of economic globalization, construction cost has become the focus of attention in the construction industry [10]. The most direct effect of the serious waste and low productivity caused by the lagging application of information technology in the construction industry is reflected in the cost [11]. Thus, how to use modern science and technology to reasonably control the cost of construction has become an urgent task for the construction industry. For a long time, experts and scholars at home and abroad have been committed to research the whole process of cost management of construction projects. They hope that, through the continuous management and control of the whole process of construction project cost, the organic link between each stage can be established, so as to realize the whole process and all-round project cost management [12–14]. However, at present, there is still a clear division of labor between design and construction in China's construction industry, and the vast majority of construction project management models adopt the DBB (Design, Bid, and Build) model. This model can hinder the transmission of information between the various stages of engineering construction and poor communication between the parties involved, making it difficult to implement whole process cost management for construction projects [15]. In this context, people began to explore a large number of new technologies and management models, such as three-dimensional mapping [16], lean construction management model [17], integrated project delivery management model [18], BIM model [19], system dynamic model [20], and electroencephalogram model [21], the purpose of which is to build bridges between the various stages of the project and the various parties involved, so as to reduce information communication obstacles, so that the whole process of construction project cost management can be implemented. Currently, 3D graphics technology has been implemented in the budgeting software, allowing users to quickly implement 3D graphics modeling in the budgeting software, and the system will automatically calculate and summarize the quantity information and establish a link with the cost estimation database to generate cost estimates. The system will automatically calculate the summary quantity information and establish a link with the cost estimate database to generate cost estimates [22]. This model supports the construction of geometric operations and spatial topology relationships, which not only improves the accuracy of cost estimates but also improves intelligence and automation [23]. However, there are several problems with this technology. For instance, there is not yet a good interface between cost forecasting and design, which prevents the direct use of design information and affects the efficiency of cost forecasting and control. Furthermore, there is no intuitive link between building components and cost information, and when design changes or changes in cost parameters occur, they need to be adjusted item by item in each system, which is less efficient.

With the rapid development of information technology today, information resources have become one of the three strategic resources in the new century. Along with the continuous development of Internet technology and the data industry, the capacity and performance of computer hardware have also been upgraded, which has made it possible for the development of information technology in all industries [24]. In China, the level of informatization in the electronics and manufacturing industries is already at a high level, and the models generated by information technology have significantly improved the productivity of these industries, but the informatization of the construction industry is still in its infancy. Informatization in the construction industry refers to the use of information technology, such as computer technology, communication technology, control technology, and information security technology, to transform and upgrade the technical means and production organization of the construction industry, thereby improving the management level and core competitiveness of construction enterprises. In developed countries such as Europe and the USA, where information technology in the construction industry started earlier, the application of information technology in construction enterprises has become more common, mainly in the extensive use of BIM [25]. BIM is a new concept that has emerged in the construction industry in recent years. Its introduction and development have placed higher demands on decision-makers and participants in engineering and construction projects in terms of collaborative work and application management for information sharing. In terms of modeling, BIM is based on three-dimensional digital technology, with the database formed by the three-dimensional model as the core [26]. The whole modeling process not only contains the professional design concepts of designers from various disciplines but also contains information on the whole process from design to construction and even completion and final demolition. From the application point of view, the model is based on the parametric design and is an object-oriented, parametric, and intelligent digital representation of the building, supporting various operations in construction projects and containing engineering information that is all interrelated [27]. Based on the parametric, visual, collaborative, and information-sharing advantages of BIM models, BIM can facilitate the early involvement of all parties involved in construction projects. In addition, the application of BIM can help constructors make the most effective decisions and use BIM to participate in construction project management during the construction process, thereby reducing project duration, controlling project risks, and promoting the smooth implementation of the whole process cost management.

The understanding of what BIM means varies due to the different levels of understanding of BIM. The most straightforward understanding of BIM is that it is a production tool that is used as a platform on which all information related to a construction project is placed to create a building model that simulates the real situation of the building [28]. BIM is also a new management concept, as the building model created through BIM can accommodate information from all stages of the building construction process and can be updated at any time to facilitate the participation and cooperation of all parties involved in the project, thus achieving whole life cycle management from design to construction, operation, and even demolition. With the support of BIM technology, all parties involved in the project can visualize the results of the design through BIM software. With the help of BIM, communication between the various project participants is made easier. Through construction simulations, they can identify any design irregularities in advance, thus reducing the need for rework due to changes and optimizing the design [29]. Cost management before the actual construction of the project can prevent the actual cost of the project from exceeding the budget after completion. As a result, the emergence and application of BIM technology provide a reference for the realization of whole process cost management [30]. However, there are still a few cases of BIM being used in construction projects in China, but research on BIM has already started. Some universities have set up BIM research groups to research BIM theory, BIM software, and BIM applications [31]. The research and development of BIM and the use of BIM technology to build an information platform to facilitate the efficient implementation of the whole process of construction project cost management will be a key focus of future research and development in the field of construction project costing in China.

In this context, this paper investigates the whole process of cost management of project construction based on BIM technology, focusing on the whole process of cost control of engineering construction projects from conceptual design to construction and completion and handover. In addition, this research focuses on the application of BIM technology in the whole process cost management of construction projects as an example and proposes a proven solution for BIM technology in the whole process cost management of construction projects.

## 2. Construction Cost Management

The construction cost refers to the construction price of an item of engineering construction. In a broad sense, construction cost covers construction cost, installation cost, municipal cost, power cost, water cost, and communication cost. The meaning of construction cost can be understood in two different ways from the perspective of the owner and the contractor, respectively. From the owner's point of view, the construction cost is the total one-off cost of the planned reproduction of fixed assets, the formation of corresponding intangible assets, and the laying down of working capital, i.e., the investment in the fixed assets of the construction project. On the other hand, from the contractor's point of view, the construction cost is the price of the construction and installation work and the total price of the construction work expected or formed in the land market, the technical labor market, and the equipment market, and other trading activities.

### 2.1. Current Status of Construction Cost Management in China

**Figure 1:**
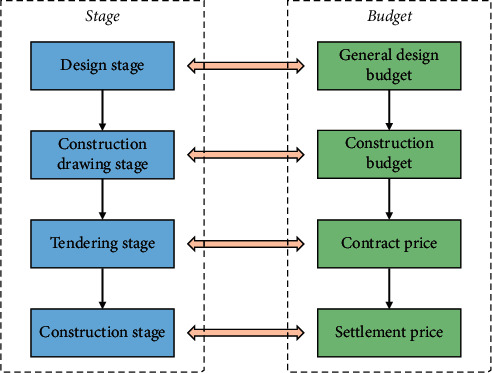
Construction stage and its corresponding budget estimates.

At the present stage, China's construction cost management mode is a whole process construction cost management mode in which fixed-price pricing and bill-of-contract pricing coexist. The whole process of cost management refers to reasonable determination and effective control of project costs throughout the entire process from the decision-making stage to the completion and acceptance of the project. To facilitate the establishment of economic relations between the parties in the process of construction and to meet the requirements of construction management, budget estimates are required for each stage of construction. Each construction stage and its corresponding budget estimates are shown in Figure 1.

In recent years, with the improvement of the bidding system and the continuous development of the quota standard, the cost management level of China's construction industry has been significantly improved, and cost consultancy has become a relatively mature profession. However, the level of cost management of construction projects in China still has a large gap with that of developed countries, and the current situation of cost management of construction projects in China is not optimistic. From the perspective of project cost management, each stage of cost management can reflect common features. The relationship between the various stages of a construction project also inevitably requires that the cost management of each stage is also coherent with each other. The entire construction project cost management should form an organic whole, so that all parties involved in the construction project can be the first to know the occurrence and changes in construction investment and cost. Therefore, the whole process of cost management of construction projects is the most advantageous management mode at present. At the same time, the current situation of cost management in China's construction projects and many problems that have been revealed have made whole process cost management an inevitable trend.

### 2.2. Building Lifecycle Management

Building lifecycle management (BLM) is a digital approach to creating, managing, and sharing information about the capital assets of a project throughout its construction. The core idea is to integrate the design, construction, and management processes through information integration and collaborative working. BLM covers all aspects of the full project lifecycle, including component design, document management, cost forecasting, construction management, project management, and visualization in the decision phase, implementation phase, and operational phase. The construction information is characterized by the large volume, diversity, and frequent changes, and the difficulty of managing project information is exacerbated by the large number of parties involved. The starting point of BLM is to solve the challenges of project information management, including information creation, management, and sharing. The process of creating information involves the selection of solutions and the integration of relevant information to ensure the accuracy of the information, including project solutions, spatial geometric properties, bills of materials, product structures, and cost information. An important way to achieve the creation of information is through the use of BIM technology.

**Figure 2:**
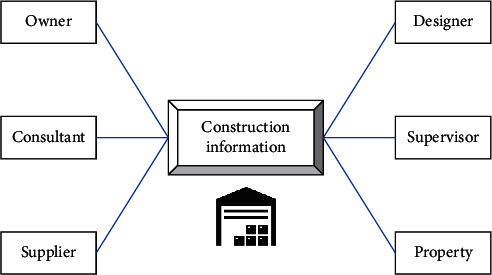
Framework of BLM.

The realization of the concept needs to rely on the support of relevant technical software. As shown in Figure 2, BIM technology completes the collection and creation of basic building information data, and each participant extracts and uses project information through their respective data interfaces to realize the value-added final realization of the project concept.

**Figure 3:**
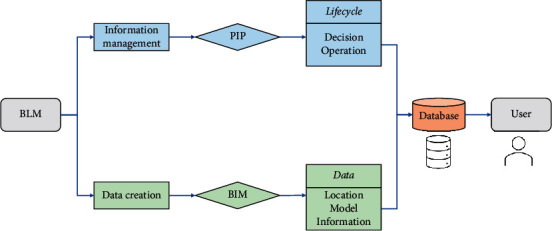
Realization process of BLM.

The core purpose of BLM technology is to solve the problem of information creation, information management, and information sharing in the whole life cycle of a construction project, and the process of its realization can be expressed in Figure 3.

### 2.3. Building Information Modeling

Building information modeling (BIM) is the process of creating and managing building information. It is a technique for modeling the entire construction project through one or more building information databases and is a parametric model containing a variety of information. Through technology-based design software, designers can input parametric information directly into the database in a graphical environment, without the need to expend more effort on abstract two-dimensional drawings, to obtain objects with a range of characteristics to represent the basic properties of a building. In recent years, with the widespread application of modern computer technology in the construction industry, BIM technology has slowly been realized. The application and promotion of BIM technology is not only a technical upgrade to the construction industry but also a revolution to the traditional concept of construction engineering.

**Figure 4:**
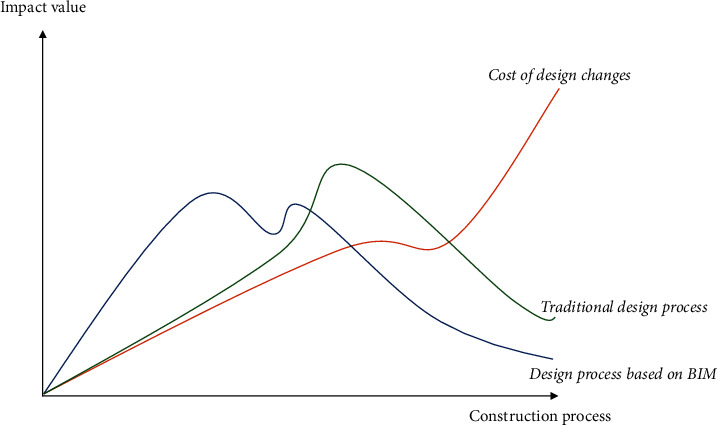
Difference between design process based on BIM and traditional design process.

With the support of BIM technology, designers can carry out a comparative analysis of multiple solutions, thus minimizing the impact of design changes or professional conflicts on construction projects and providing security for project decisions. Designers can directly create parametric 3D building models through BIM-related software, putting more effort into the design rather than wasting a lot of time on 2D output drawings.  Figure 4 reflects the difference between the design process based on BIM and the traditional design process.

**Figure 5:**
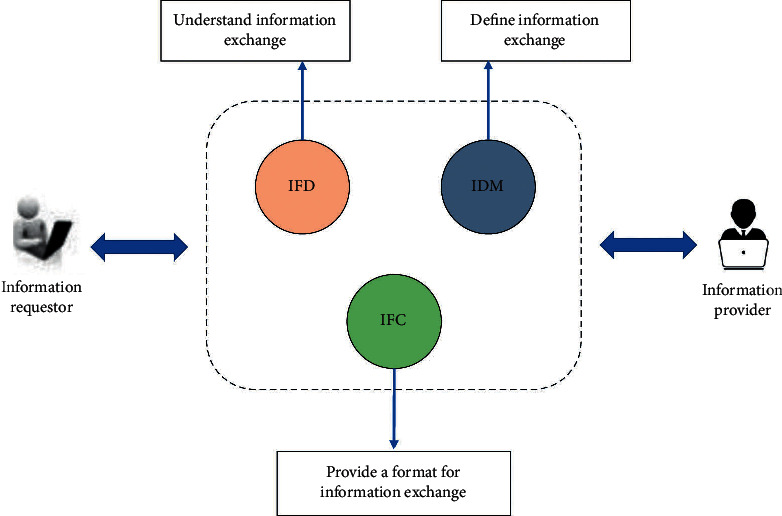
Information exchange among IFC, IDM, and IFD.

The key to BIM applications is how to solve the problem of information expression, information transfer, and information exchange. For a long time, it has been difficult to exchange information between different software due to the large differences in data formats between them. To solve this problem, the most effective way is to develop a data file standard that all software can support so that all software can exchange information with each other through this data file standard. For this reason, a number of standards have been implemented internationally to regulate the representation and exchange of different data, and three of the more popular ones are industry foundation class (IFC), information delivery manual (IDM), and international framework for dictionaries (IFD). As shown in Figure 5, IFC, IDM, and IFD form the basis for information exchange on the BIM platform, ensuring the smooth transfer and sharing of information on construction projects and thus maximizing the value of the BIM technology platform. IDM is the process of defining the types of information that need to be exchanged between different phases and objects and the methods of exchange and ensuring that these information exchange processes are properly understood and used. The relationship between FC and IDM can be understood as the IFC is like a pharmacy with all the medicines, while the IDM is a prescription for a particular disease or a particular patient. That is, the IFC supports all business requirements between all projects and all phases, while the IDM supports one business requirement for one project and one phase, and it is the IDM that decides which IFC information is needed for this business.

## 3. BIM-Based Construction Cost Management

The first step in cost budgeting is quantity surveying. The biggest advantage of building information model quantity surveying over traditional drawing surveying is that it minimizes the need for manual surveying.

### 3.1. Investment Decision Stage

**Figure 6:**
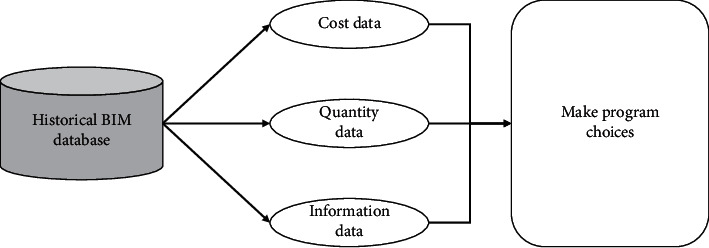
Program choice based on BIM.

The investment decision stage is the most crucial step in the construction of a project, where different investment options are economically and technically justified, and the best option is selected after comparison. A mistake in decision-making can often bring irreparable losses to the enterprise and even plunge it into an economic crisis, so the investment decision stage of the project needs to be given high priority. The content of the investment decision stage is the basis for determining the cost of the project, and a correct investment decision requires an accurate grasp of the costs of each option. Therefore, on the premise of technical feasibility, it is essential to make investment estimates for each option. The use of Excel archives has been a further development, but for many reasons, the amount of data that can be accumulated is small. Historical data are less structured, less calculable, and more cumbersome to accumulate. The BIM model has construction data, technical data, quantity data, cost data, schedule data, and application data that can be restored when comparing and selecting investment options and can be displayed in a three-dimensional mode. As shown in Figure 6, the model of a project with a similar history can be changed and innovated according to the program characteristics of the new project, so that the cost and total amount of work can be calculated for several costs programs. Based on this procedure, the selection of the overall solution is made easier, making it much more efficient and helping to design future solutions.

### 3.2. Design Stage

The preparation of estimates at the design stage depends on the depth of the design, the degree of completeness of the information, and the requirements for the accuracy of the estimates. When design information is insufficient, budgets for similar projects can be selected as a basis for preparation after analysis and adjustment of coefficients. If the information on similar projects is not available, the budget estimates will be prepared using indicators. When the design has reached a certain depth and a detailed list of equipment, a sketch of the pipeline alignment, building and structure type, and technical requirements for construction can be provided, and the estimate is prepared on the basis of quotas and cost indicators.

**Figure 7:**
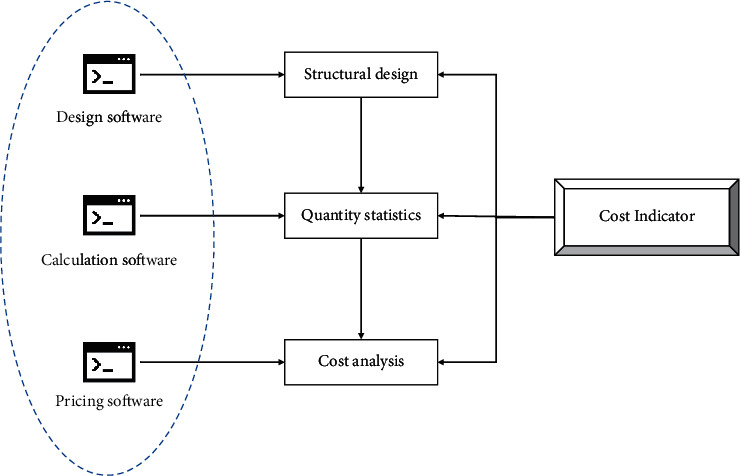
Construction limit design process.

It is now generally accepted that cost management at the design stage should focus on limit design, i.e., the initial scheme design should be based on investment estimates. Nowadays, it is not easy to achieve a reasonable limit design based on traditional manual algorithms and engineering budgeting methods. Firstly, the varying technical levels of the designers and the lack of cost control thinking mean that the tasks of the different disciplines are separate, and coordination and control have to be carried out regularly. Furthermore, the lack of adequate cost information in the design drawings due to the current design approach means that cost consultancy work and design work cannot be synchronized, and design proposals cannot be revised in a timely manner due to the constraints of cost indicators. As shown in Figure 7, with the introduction of BIM technology, the designer can extract some of the relevant design indicators from the model database for a more rapid limit design, thus achieving the goal of an economical and reasonable design. Along with this objective, construction information and the corresponding quantity information can be obtained by the cost engineer and compared with the information in the database, so that the estimated price can be derived more quickly. The reasonableness of the design indicators is then verified, and the value engineering approach is combined with the control of construction and use costs based on the whole project cycle, thus optimizing the design solution. The BIM model's real-time modeling and accounting of costs can be used as a basis for designers and cost engineers to carry out real-time and simultaneous calculations and analyses of the cost of the units designed, so that the information obtained can be used to optimize every detail of the design scheme, thus enabling limit design to be achieved.

**Figure 8:**
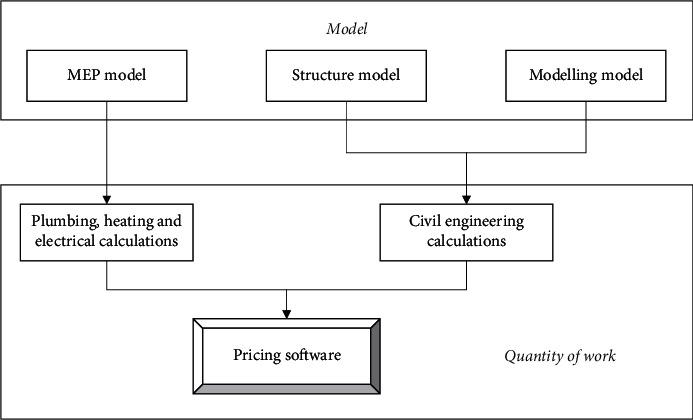
Interface relationship between design and calculation software.

The establishment of a great BIM model is highly valued by engineering cost practitioners, different professions have different BIM models, and the quality of the BIM model has a direct impact on the outcome of the project.  Figure 8 illustrates the interface relationship between design and calculation software. Many domestic costing-related software companies are doing their best to develop relevant model data interfaces to implement the model design. Their efforts have been tested in several engineering cases, and some results have gradually been achieved. With the promulgation of national standards for the interface from design to measurement, this technology is gradually being accepted by engineers.

## 4. Conclusion

In the current construction industry, there are many participants and a huge amount of information. Traditional construction cost management methods and information communication mechanisms have been unable to meet the requirements of modern construction project cost management work and must be committed to exploring new ways of working and information transmission channels to realize the whole process of construction project cost management. Only in this way can China's current construction project cost management be optimized, the overall cost control level of the industry be improved, and the waste of energy and resources be reduced, so that we can ultimately stand out in the fierce international competitive environment. In order to realize the whole process of cost management of construction projects, this study applies BIM technology to it. BIM is suitable for the whole process of cost management in construction projects. It can be used to effectively improve the efficiency of cost management at all stages and control the total cost of construction projects. BIM establishes an information-sharing platform to share information and improve the efficiency of cost management at each stage. In addition, BIM technology can achieve coordination and cooperation in cost management between all stages and all parties involved, thus realizing BIM-based cost management for the whole construction process, which is the key to solving the current problems of cost management for the whole construction process in China.

However, this paper is only a theoretical study and discussion of how to achieve BIM-based construction cost management, and there are still the following problems to be solved. Firstly, further research should be carried out on the creation of a computer-literate API interface to BIM software data and a database of BIM correspondence with lists and quotations. In addition, it is necessary to explore how to establish working methods and communication mechanisms between the various stages of the project and between the various parties involved in specific business operations based on BIM.

## Data Availability

The labeled datasets used to support the findings of this study are available from the corresponding author upon request.
